# TXNIP regulates AKT‐mediated cellular senescence by direct interaction under glucose‐mediated metabolic stress

**DOI:** 10.1111/acel.12836

**Published:** 2018-08-31

**Authors:** Hangsak Huy, Hae Young Song, Mi Jeong Kim, Won Sam Kim, Dong Oh Kim, Jae‐Eun Byun, Jungwoon Lee, Young‐Jun Park, Tae‐Don Kim, Suk Ran Yoon, Eun‐Ji Choi, Chul‐Ho Lee, Ji‐Yoon Noh, Haiyoung Jung, Inpyo Choi

**Affiliations:** ^1^ Immunotherapy Convergence Research Center Korea Research Institute of Bioscience and Biotechnology (KRIBB) Daejeon Korea; ^2^ Department of Functional Genomics University of Science and Technology (UST) Daejeon Korea; ^3^ Department of Biochemistry, School of Life Sciences Chungbuk National University Cheongju Korea; ^4^ Department of Hematology, Asan Medical Center University of Ulsan College of Medicine Seoul Korea; ^5^ Laboratory Animal Resource Center Korea Research Institute of Bioscience and Biotechnology (KRIBB) Daejeon Korea

**Keywords:** aging, AKT, glucose, reactive oxygen species, TXNIP

## Abstract

Aging is associated with an inevitable and universal loss of cell homeostasis and restricts an organism's lifespan by an increased susceptibility to diseases and tissue degeneration. The glucose uptake associated with producing energy for cell survival is one of the major causes of ROS production under physiological conditions. However, the overall mechanisms by which glucose uptake results in cellular senescence remain mysterious. In this study, we found that TXNIP deficiency accelerated the senescent phenotypes of MEF cells under high glucose condition. TXNIP^‐/‐^ MEF cells showed greater induced glucose uptake and ROS levels than wild‐type cells, and *N*‐acetylcysteine (NAC) treatment rescued the cellular senescence of TXNIP^‐/‐^ MEF cells. Interestingly, TXNIP^‐/‐^ MEF cells showed continuous activation of AKT during long‐term subculture, and AKT signaling inhibition completely blocked the cellular senescence of TXNIP^‐/‐^ MEF cells. In addition, we found that TXNIP interacted with AKT via the PH domain of AKT, and their interaction was increased by high glucose or H_2_O_2_ treatment. The inhibition of AKT activity by TXNIP was confirmed using western blotting and an in vitro kinase assay. TXNIP deficiency in type 1 diabetes mice (Akita) efficiently decreased the blood glucose levels and finally increased mouse survival. However, in normal mice, TXNIP deficiency induced metabolic aging of mice and cellular senescence of kidney cells by inducing AKT activity and aging‐associated gene expression. Altogether, these results suggest that TXNIP regulates cellular senescence by inhibiting AKT pathways via a direct interaction under conditions of glucose‐derived metabolic stress.

## 1 INTRODUCTION

Cells are constantly exposed to metabolic stress, a major cause of cellular senescence. Recent reports have shown that metabolic changes influence aging in model systems, from the budding yeast to mouse models (Gelino & Hansen, 2012). One of the prominent cellular senescence markers is the accumulation of reactive molecules, such as reactive oxygen species (ROS), a product of an essential energy production (Rando & Chang, 2012). Glucose serves as an energy source in virtually all eukaryotic cells. A high concentration of glucose increases the metabolic input into cells and consequently induces oxidative stress via ROS production, thereby inducing DNA, protein, and lipid damage, causing premature senescence (Mortuza, Chen, Feng, Sen, & Chakrabarti, 2013; Simone, Gorin, Velagapudi, Abboud, & Habib, 2008).

Thioredoxin‐interacting protein (TXNIP) was originally identified in 1994 as a gene upregulated in HL‐60 cells treated with vitamin D3 (Jung & Choi, 2014). TXNIP is an α‐arrestin family protein that is induced by a rise in glucose and oxidative stress and is known to be a tumor suppressor and inhibit thioredoxin (TRX), an antioxidant protein, via a direct interaction (DeBalsi et al., 2014; Jung et al., 2016, 2013 ). Many studies have examined the role of TXNIP in glucose uptake and metabolism. TXNIP was shown to suppress glucose uptake by regulating glucose transporter 1 (GLUT1) localization and expression (Wu et al., 2013) and peripheral glucose metabolism (Parikh et al., 2007), and its expression in the liver is required for maintaining normal fasting glycemia and glucose production (Chutkow, Patwari, Yoshioka, & Lee, 2008). TXNIP regulates glycolysis and cell growth by reductive reactivation of phosphatase and tensin homolog (PTEN), and TXNIP deficiency results in protein kinase B (AKT) activation in oxidative tissues (Hui et al., 2008). TXNIP expression is related to mitochondrial fuel switching under conditions of starvation, diabetes, and exercise in skeletal muscle (DeBalsi et al., 2014). Previously, we suggested that TXNIP is highly expressed and acts as an antioxidant protein to regulate cellular ROS by activating p53 activity or by inhibiting p38 mitogen‐activated protein kinase (MAPK) activity via direct interaction in hematopoietic stem cells (HSCs) (Jung & Choi, 2014; Jung et al., 2016, 2013 ). Importantly, TXNIP‐deficient mice are more glucose‐tolerant than wild‐type (WT) mice (Hui et al., 2008).

AKT is a serine–threonine kinase that is involved in a variety of cellular processes including cell survival, proliferation, and metabolism (Hsieh, Lin, Bennett, Wu, & Wu, 2014). AKT has a structure consisting of an N‐terminal pleckstrin homology (PH) domain, a kinase domain (KD), and a C‐terminal hydrophobic regulatory region (HM) (Parikh et al., 2012). Extensive studies have reported the necessity of AKT in the regulation of glucose uptake and metabolism. AKT plays an essential role in the insulin‐regulated transport of glucose and in whole‐body glucose homoeostasis via an AKT‐dependent movement of GLUT4 to the plasma membrane (Tan, Ng, & James, 2010), and activation of the AKT pathway is directly correlated with increased rates of glucose metabolism (Hajduch, Litherland, & Hundal, 2001). The phosphoinositide 3‐kinase (PI3K)/AKT pathway promotes cellular glucose uptake by downregulating the expression of TXNIP (Hong, Yu, Luo, & Hagen, 2016). The activation of AKT induces intracellular ROS by inducing oxygen consumption or inhibiting the forkhead box O (FOXO) family of transcription factors, in turn, promoting cellular senescence and apoptosis (Dolado & Nebreda, 2008; Nogueira et al., 2008). AKT also activates the mechanistic target of rapamycin (mTOR) and induces cellular senescence (Johnson, Rabinovitch, & Kaeberlein, 2013). Furthermore, high glucose levels stimulate protein synthesis and induce ROS production by AKT phosphorylation (Sheu, Ho, Chao, Kuo, & Liu, 2004; Simone et al., 2008).

In this study, we found that TXNIP deficiency induces accelerated senescent phenotypes of mouse embryonic fibroblast (MEF) cells under high glucose condition and that the induction of cellular ROS or AKT activation is critical for cellular senescence. Our results also revealed that TXNIP inhibits AKT activity by a direct interaction via the PH domain of AKT, which is upregulated by high glucose and H_2_O_2_ treatment. In addition, TXNIP^‐/‐^ mice exhibited an increase in glucose uptake and aging‐associated phenotypes including a decrease in energy metabolism and induction of cellular senescence and aging‐associated gene expression. We propose that TXNIP is a critical regulator of AKT‐mediated cellular senescence under glucose‐mediated stress in vitro and in vivo.

## 2 RESULTS

### 2.1 Accelerated senescent phenotypes in TXNIP^‐/‐^ cells

**Figure 1:**
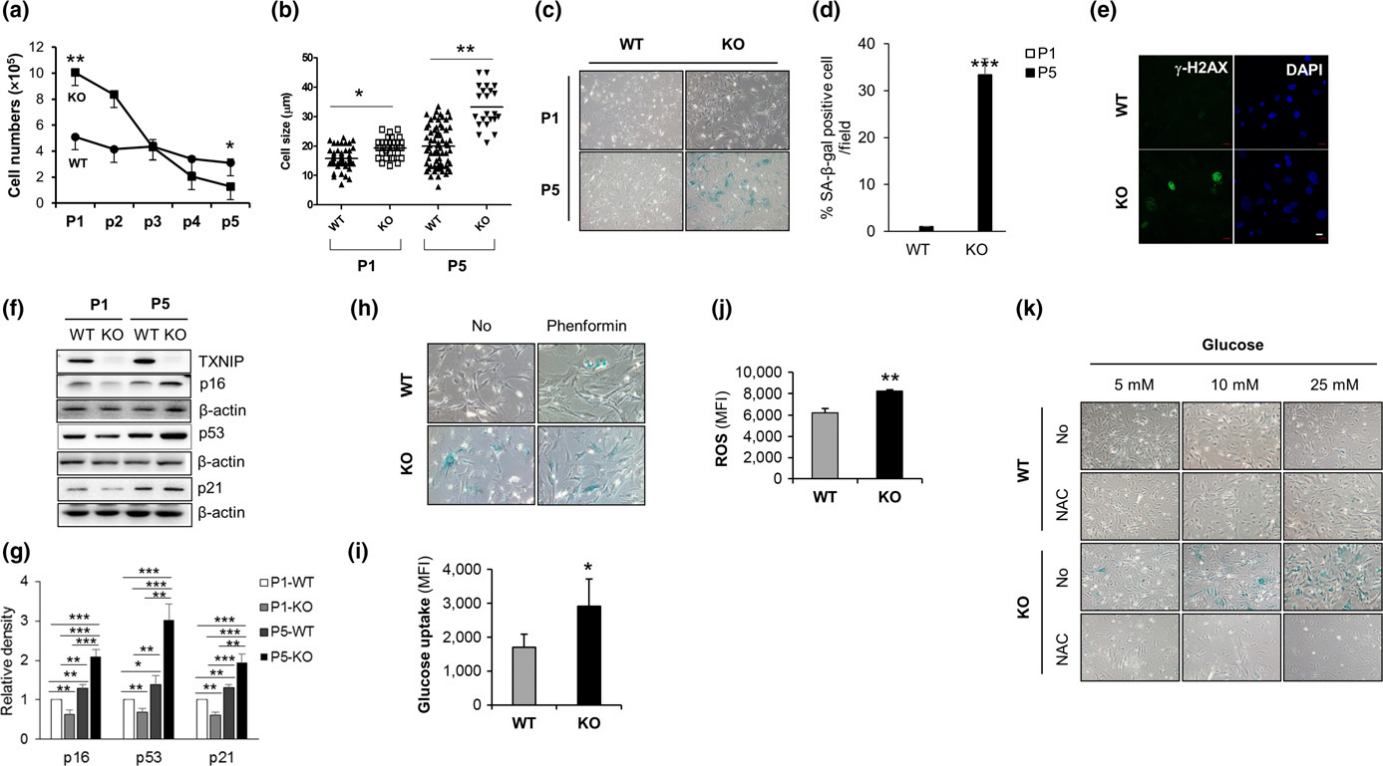
High glucose induces senescence of KO MEF cells. (a) WT and KO MEF cells were initially plated at 2 × 10^5^ cells and cultured by subculturing at 3‐day intervals and the cell number counted at the same intervals or same passages (from P1 to P5) (*n* = 3). (b) The size of trypsinized MEF cells was measured for the longest diameter by microscopic images. (c) Representative images of SA‐β‐gal staining at Passage 1 (P1) and Passage 5 (P5) (*n* = 5). (d) SA‐β‐gal staining in (c). Stained cells were counted by % of captured total MEF cells (*n* = 5). Magnification: 200X. (e) Representative confocal images of γ‐H2AX‐stained MEF cells at P5. White scale bar indicates 20 µm. Repeated four times. (f, g) The expression of cell cycle regulators in MEF cells. Western blotting (f) and densitometric analysis (g). Repeated three times. (h) Representative images of SA‐β‐gal staining of WT and KO MEF cells at P5. MEF cells were daily treated with or without 1 mM phenformin and continuously subcultured at 3 days intervals from P1 to P5. (i) Glucose uptake of MEF cells at P5. MEF cells were incubated with 2‐NBDG (30 µg/ml) for 20 min, and MFI was determined using flow cytometry (*n* = 4). (j) ROS levels in MEF cells at P5 (*n* = 4). (k) Representative images of SA‐β‐gal staining of MEF cells at P5. MEF cells were subcultured with various doses of glucose and with or without NAC (1 mM). Data are mean ± *SD*. Statistical significance was determined using Student's *t* tests. *p < 0.05, **p < 0.01, ***p < 0.001

For the senescence‐dependent proliferation assay, we prepared wild‐type (WT) and TXNIP^‐/‐^ (KO) MEF cells, which undergo senescence after a limited number of passages in culture (Kuilman, Michaloglou, Mooi, & Peeper, 2010). WT and KO MEF cells were cultured by subculturing 2 × 10^5^ cells at 3‐day intervals to investigate the function of TXNIP on replicative senescence (Dimri et al., 1995), with the cell number counted at the same intervals. Surprisingly, KO MEF cells proliferated at a higher rate than WT MEF cells at early passages (P1 and P2); however, the growth rate of KO MEF cells was dramatically decreased, and the cells almost ceased to proliferate at late passages (P5) (Figure 1a). Previous reports have suggested that senescent cells are larger than nonsenescent cells, are stained by senescence‐associated beta‐galactosidase (SA‐β‐gal), and showed the increased activity of γ‐H2AX, the phosphorylation form of histone H2A member X at serine 139 (Dimri et al., 1995; He et al., 2016). To evaluate the cellular senescence, we examined the size of MEF cells and performed SA‐β‐gal assays and confocal imaging of γ‐H2AX. KO MEF cells were larger than WT MEF cells (Figure 1b). KO MEF cells were stained with SA‐β‐gal and γ‐H2AX at Passage 5 (P5) (Figure 1c‐e). Next, to confirm the cellular senescence of KO MEF cells, we examined the expression of senescence‐associated genes including p53, p21, p16, PAI‐1, and PML. The expression levels were determined by western blotting and statistical analysis (Figure 1f,g and Supporting Information Figure S5) or quantitative real‐time PCR (Supporting Information Figure S1A,B). We previously reported the regulation of p53 by TXNIP (Jung et al., 2013). To examine the effects of p53 on the cellular senescence, we prepared WT, KO, p53^‐/‐^, and TXNIP^‐/‐^p53^‐/‐^ (DKO) MEF cells. Interestingly, p53 deficiency in KO MEF cells completely blocked the cellular senescence of KO MEF cells at P5 (Supporting Information Figure S1C,D). In addition, TXNIP deficiency also markedly induced cellular senescence of the lung fibroblasts at P5 (Supporting Information Figure S1E). These results suggest that TXNIP regulates cellular senescence of MEF cells. Glucose availability affects ROS production in cells, and high glucose uptake induces cellular senescence (Mortuza et al., 2013). Previous reports have revealed that TXNIP regulates glucose uptake in peripheral tissues in both an insulin‐dependent and insulin‐independent manner and suppresses glucose uptake by regulating the expression and internalization of GLUT1 (Parikh et al., 2007; Wu et al., 2013). From these previous reports, we hypothesized that TXNIP might regulate cellular senescence by regulating the balance of glucose uptake and ROS production. To induce cellular senescence, we treated MEF cells with glucose uptake‐inducing agent, phenformin, which is known diabetes drug. As expected, continuous treatment from P1 to P5 with phenformin induced cellular senescence of WT MEF cells at P5 (Figure 1h and Supporting Information Figure S1G). Next, we examined the expression of TXNIP in WT MEF cells treated with phenformin at various doses. It dramatically suppressed the level of TXNIP (Supporting Information Figure S1H), induced glucose uptake (Supporting Information Figure S1I), suppressed cell proliferation (Supporting Information Figure S1J), and induced the level of p16 (Supporting Information Figure S1K) in WT MEF cells. As previously reported, phenformin has multiple mode of action including the extremely well‐documented activation of AMPK. Furthermore, sustained activation of AMPK is not only a hallmark of senescence but is also sufficient to trigger cellular senescence (Jiang, Du, Mancuso, Wellen, & Yang, 2013; Jones et al., 2005). From these results, we could not exclude the possible roles of TXNIP in AMPK‐induced cellular senescence under phenformin treatment condition. As previously reported (H. Parikh et al., 2007), KO MEF cells showed higher glucose uptake at P1 (Supporting Information Figure S1L) and P5 (Figure 1i) and overexpressed TXNIP suppressed glucose uptake in WT MEF cells (Supporting Information Figure S1M). To demonstrate the glucose uptake‐induced ROS production in MEF cells, we determined the levels of ROS at P5 of WT MEF and KO MEF cells. KO MEF cells showed higher levels of ROS (Figure 1j). Next, to examine the direct effects of glucose uptake and ROS on cellular senescence of KO MEF cells, we treated MEF cells with various concentrations of glucose alone or in combination with *N*‐acetyl cysteine (NAC), an antioxidant agent. Interestingly, the SA‐β‐gal staining of KO MEF cells gradually increased with glucose treatment in a dose‐dependent manner; however, NAC treatment completely blocked the senescence of KO MEF cells at all concentrations of glucose (Figure 1k and Supporting Information Figure S1N). Taken together, our results imply that TXNIP regulates cellular senescence by regulating glucose uptake and ROS production in MEF cells.

### 2.2 AKT‐mediated senescence of KO MEF cells

**Figure 2:**
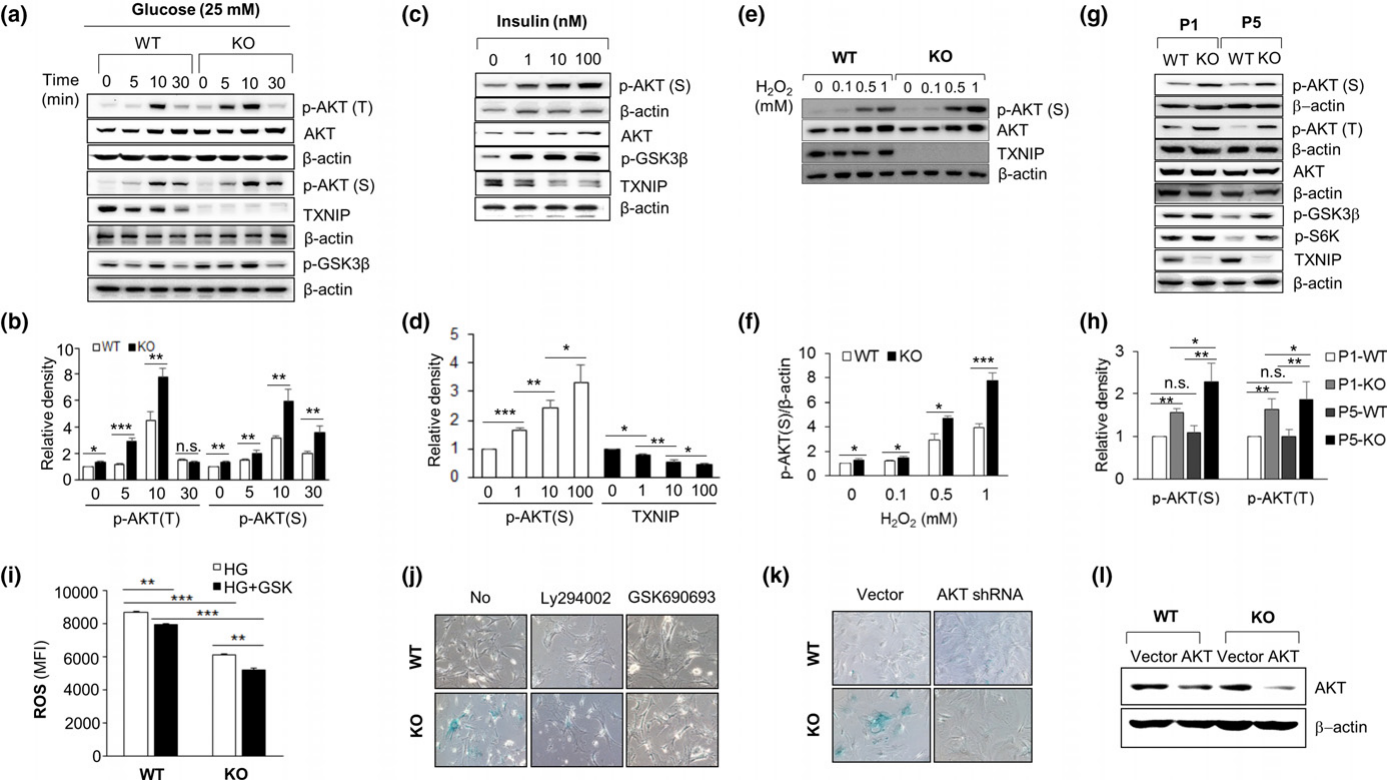
The activation of AKT in KO MEF cells induces cellular senescence. (a) AKT is differentially activated by high glucose (25 mM) treatment in KO MEF cells. Repeated three times. (b) Quantification of active AKT in (a). Repeated three times. (c) The activation of AKT and the reduction in TXNIP by insulin treatment. MEF cells were treated with insulin for 1 hr. Repeated three times. (d) Statistical analysis of p‐AKT(S) and TXNIP levels in (c). Repeated three times. (e, f) AKT is differentially activated by H_2_O_2_ in KO MEF cells. Repeated three times. (g, h) Activation of AKT in KO MEF cells at P1 and P5. Repeated three times. (i) AKT inhibitor, GSK690693, inhibits the induction of ROS in P1 MEF cells under high glucose condition for 24‐hr treatment (*n* = 4). (j) SA‐β‐gal staining of KO MEF cells is blocked by PI3K inhibitor or AKT inhibitor treatment at P5. MEF cells were subcultured from P1 to P5 and treated with DMSO or LY294002 (20 µM) or GSK690693 (5 µM) every 3 days (*n* = 5). (k) AKT shRNA blocks SA‐β‐gal staining of KO MEF cells at P5 (*n* = 5). Transduced MEF cells were subcultured from P1 to P5. (l) Knockdown of AKT in MEF cells by AKT shRNA at P5. Repeated two times. Data are mean ± *SD*. Statistical significance was determined using Student's *t* tests. *p < 0.05, **p < 0.01, ***p < 0.001

One of the emerging effectors of intracellular ROS production and replicative senescence under metabolic stress is AKT signaling. Recent reports have suggested that high glucose‐induced AKT signaling increases ROS levels by activating mTOR signaling, which induces glucose metabolism and protein synthesis, and by inhibiting FOXO signaling, which induces antioxidant gene transcription (Nogueira et al., 2008; Sheu et al., 2004). Consequently, AKT activation increases metabolic stress and ROS, resulting in a positive feedback loop that promotes cellular senescence (Dolado & Nebreda, 2008; Zhao et al., 2017). To investigate the activation of AKT in WT and KO MEF cells, we treated MEF cells with various concentrations of glucose or insulin, a peptide hormone that regulates glucose uptake, as a metabolic stress inducer. AKT was activated by high glucose treatment, and its activation in KO MEF cells was comparable to that in WT MEF cells (Figure 2a,b and Supporting Information Figure S5). AKT was also activated by insulin in a dose‐dependent manner (Figure 2c,d and Supporting Information Figure S5). Interestingly, the level of TXNIP was negatively associated with AKT activation in both the glucose and insulin treatment conditions. To prove the regulatory mechanism of TXNIP on AKT activation under oxidative stress, we treated MEF cells with H_2_O_2_. AKT was highly activated by H_2_O_2_ in KO MEF cells in a dose‐dependent manner (Figure 2e,f and Supporting Information Figure S2A). We also examined the activation of AKT in MEF cells at P1 and P5 and found that AKT was highly activated in KO MEF cells at both passages (Figure 2g,h and Supporting Information Figure S5). Next, to determine the function of AKT on cellular senescence, we subcultured MEF cells with LY294002, a PI3K inhibitor, or GSK690693, a specific AKT inhibitor from P1 to P5 (Cen, Mahajan, Wang, & Kraft, 2013; Tang et al., 2013). AKT inhibition decreased ROS levels in WT and KO MEF cells at P1 (Figure 2i) and suppressed glucose uptake at P5 of WT and KO MEF cells (Supporting Information Figure S2B). It also completely rescued the cellular senescence of KO MEF cells at P5 (Figure 2j and Supporting Information Figure S2C). The regulation of cellular senescence was also confirmed by an AKT knockdown experiment using RNAi (Figure 2k,l and Supporting Information Figure S2D). Overall, these results reveal that the senescence of KO MEF cells is regulated by AKT activation.

### 2.3 TXNIP regulates the activity of AKT via direct interaction

**Figure 3:**
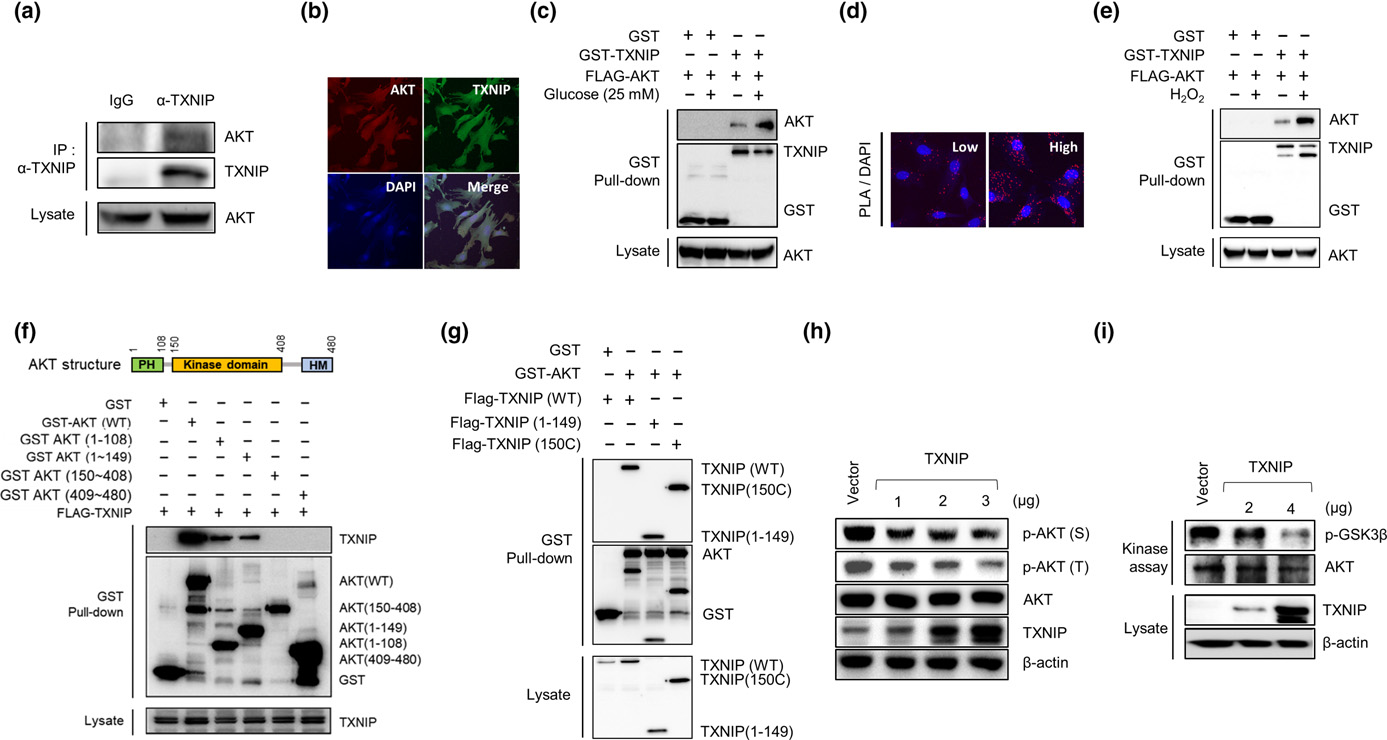
TXNIP interacts with AKT and inhibits the activity of AKT. (a) Endogenous binding between TXNIP and AKT in 293T cells. Repeated three times. (b) Confocal images of TXNIP and AKT in WT MEF cells. Repeated two times. (c) GST pull‐down assay in 293T cells. Cells were incubated with 25 mM glucose for 1 hr. Repeated two times. (d) In situ binding between TXNIP and AKT was determined using a proximity ligation assay (PLA) in MEF cells under high glucose condition. Repeated two times. (e) GST pull‐down assay in 293T cells. Cells were treated with 0.5 mM H_2_O_2_ for 1 hr. Repeated three times. (f) The interaction between TXNIP and PH domain of AKT. Repeated five times. (g) The interaction between AKT and deleted mutants of TXNIP in 293T cells. Repeated three times. (h) Overexpressed TXNIP inhibits AKT phosphorylation in MEF cells. Repeated four times. (i) In vitro kinase assay. 293T cells were transfected with TXNIP‐overexpressing plasmid, and cell lysates were immunoprecipitated and subjected to in vitro kinase assay for AKT activity. Repeated three times

**Figure 4:**
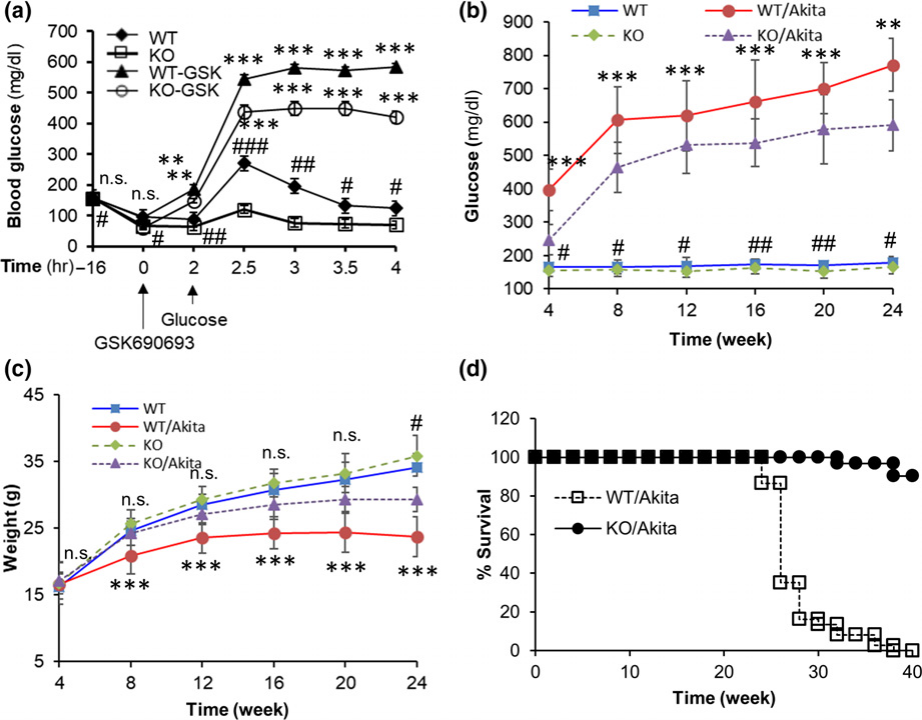
Regulation of glucose uptake by TXNIP in type 1 diabetes mice. (a) Glucose tolerance test (GTT) on fasted WT and KO mice with or without GSK690693. Mice were fasted for 16 hr and were injected with or without 30 mg/kg GSK690693 and with 2 g/kg glucose at indicated time (*n* = 5 per group). *p compared to same genotype mice and ^#^ *p* WT mice versus KO mice. (b) Blood glucose levels were measured for 24 weeks (WT: *n* = 37, Akita: 45, KO: *n* = 42, KO/Akita: *n* = 39). *p compared to KO/Akita mice and #*p* compared to KO mice. (c) Body weight of experimental mice groups (WT: *n* = 52, Akita: 56, KO: *n* = 79, KO/Akita: *n* = 45). *p compared to KO/Akita mice and #*p* compared to WT mice. (d) Mice survival assay. Mice survival is presented as a Kaplan–Meier survival curve (Akita: *n* = 37, KO/Akita: *n* = 31). * and ^#^ indicate significant difference between indicated two groups. *p < 0.05, **p < 0.01, ***p < 0.001. Statistical significance was determined using Student's *t* tests

Other groups have previously shown that TXNIP deficiency induces AKT activation via oxidative inactivation of PTEN in the soleus muscle and heart, and TXNIP is phosphorylated by AKT in response to insulin (Hui et al., 2008; Waldhart et al., 2017). To elucidate the correlation between TXNIP and AKT, we examined whether TXNIP interacts with AKT in cells. TXNIP‐ and AKT‐overexpressing plasmids were cotransfected into 293T cells, and GST pull‐down assays were performed as previously described (Kim et al., 2017, 2017). TXNIP was found to interact with AKT (Supporting Information Figure S3A), which was also confirmed by immunoprecipitation assays in 293T cells (Figure 3a). Next, to prove direct interaction between TXNIP and AKT, we performed GST pull‐down assay for recombinant proteins between GST‐TXNIP‐T (150a.a‐317a.a) and His‐AKT (Supporting Information Figure S3B). TXNIP and AKT were overlapped and expressed in cellular regions from the nucleus to cytoplasm (Figure 3b). In addition, we investigated the interaction between TXNIP and AKT under a high glucose condition. A high glucose‐induced interaction between TXNIP and AKT was determined in 293T cells using GST pull‐down assays (Figure 3c) and in MEF cells using a proximity ligation assay (PLA) (Figure 3d). The interaction between TXNIP and AKT was also induced by H_2_O_2_‐induced oxidative stress condition (Figure 3e). Next, to identify the putative interaction regions between TXNIP and AKT, we designed deletion mutants for each protein. As shown in Figure 3f, TXNIP interacted with AKT through the PH domain consisting of amino acids 1–108, which is important for AKT activation and inhibition (C. Parikh et al., 2012). AKT interacted with both deleted mutants of TXNIP (Figure 4g). These results strongly suggest that TXNIP physically interacts with AKT in cells in a glucose‐dependent manner. Next, we constructed site‐directed mutants of AKT for kinase activity‐related residues including catalytically inactive AKT (K179M) and AKT mutants with active phosphorylation sites (T308A, S473A, or T308A/S473A) (Vivanco et al., 2014). As shown in Supporting Information Figure S3C,D, the interaction between TXNIP and AKT was not affected by AKT kinase activity. Therefore, we hypothesized that TXNIP might act as an upstream regulator of AKT signaling. To examine the effect of TXNIP on AKT kinase activity, we transfected a TXNIP‐expressing plasmid into MEF cells or 293T cells. The expression of TXNIP inhibited the activity of AKT in a dose‐dependent manner in western blotting with phospho‐AKT antibodies (Figure 3h) and in vitro kinase assays (Figure 3i). Recombinant TXNIP‐T also inhibited AKT kinase activity in a dose‐dependent manner (Supporting Information Figure S3E). Taken together, these results suggest that TXNIP regulates the activity of AKT by direct interaction in cells.

### 2.4 The regulation of glucose uptake by TXNIP in vivo

To examine the effect of TXNIP or AKT on glucose uptake in vivo, we performed a glucose tolerance test (GTT) on fasted WT and KO mice (Hui et al., 2008). KO mice showed more significant glucose tolerance than WT mice from the beginning of the experiment, and glucose tolerance was significantly decreased by AKT inhibition in both WT and KO mice from 2 hr later (Figure 4a). Next, to further examine the effect of TXNIP on glucose uptake in vivo, we crossed KO mice with Akita mice, in which insulin secretion is defective (Naito et al., 2011). Akita mice showed severe and progressive hyperglycemia with time after 4 weeks of age; however, TXNIP^‐/‐^/Akita (KO/Akita) mice had significantly lower glucose levels than Akita mice at all the time points (Figure 4b). Although the body weight of both experimental groups gradually increased from birth, KO/Akita mice weighed significantly more than Akita mice from 8 weeks old (Figure 4c). TXNIP deficiency rescued the extreme hyperglycemia‐induced death observed in Akita mice (Figure 4d). These results imply that TXNIP is an important regulator of glucose uptake in vivo.

### 2.5 TXNIP deficiency decreases energy expenditure of mice

**Figure 5:**
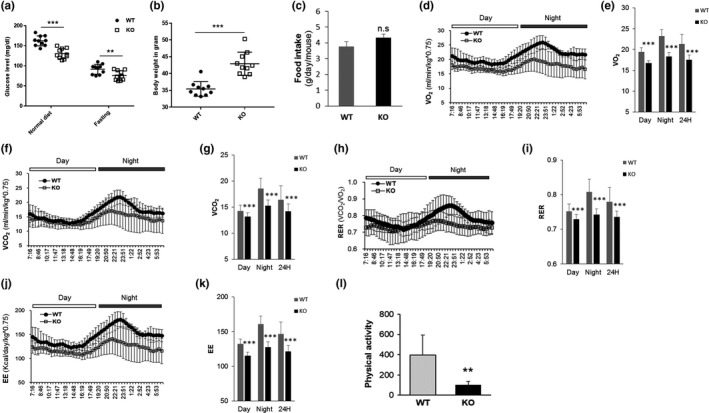
Decrease in metabolic profiles of KO mice at 12‐month‐old age. (a) Blood glucose levels of normal diet mice and fasted mice for 16 hr (WT: *n* = 10, KO: *n* = 11). (b) Body weight of WT and KO mice (*n* = 10 per group). (c) Food intake of 12‐month‐old WT and KO mice (WT: *n* = 10, KO; *n* = 10). (d, e) O_2_ consumption (VO_2_). (f, g) CO_2_ production (VCO_2_). (h, i) Respiratory exchange ratio (RER). (j, k) Energy expenditure (EE). (l) Physical activity. Twelve‐month‐old WT (*n* = 7) and KO mice (*n* = 7). Data are mean ± *SD*. Statistical significance was determined using Student's *t* tests. *p < 0.05, **p < 0.01, ***p < 0.001

As shown in Figure 4, TXNIP deficiency in mice significantly improved the features of a type 1 diabetes model. From these results, we hypothesized that TXNIP‐driven glucose uptake may be sufficient to modulate cell fate including cell death and senescence. TXNIP deficiency may induce more glucose uptake than necessary in normal cells, leading to excessive glucose supplies and increased exposure to oxidative stress over time in mice given a normal diet. Previous reports have suggested that aged mice showed less energy expenditure and physical activity than young mice (Houtkooper et al., 2011; Koonen et al., 2010). To examine the metabolic differences between WT and KO mice, we performed a metabolic analysis of 12‐month‐old WT and KO mice. The glucose levels were significantly lower in KO mice than in WT mice under normal diet and fasting conditions (Figure 5a), and KO mice weighed significantly more than WT mice (Figure 5b). Food intake was slightly higher in KO mice, but it was not statistically significant (Figure 5c). Furthermore, KO mice significantly showed lower metabolic rates in O_2_ consumption (VO_2_) (Figure 5d,e), CO_2_ production (VCO_2_) (Figure 5f,g), respiratory exchange ratio (RER) (Figure 5h,i), energy expenditure (EE) (Figure 5j,k), and physical activity (Figure 5l) than WT mice. These results suggest that TXNIP deficiency may regulate energy metabolism and physical activity in vivo.

### 2.6 TXNIP deficiency accelerates senescence of kidney cells

**Figure 6:**
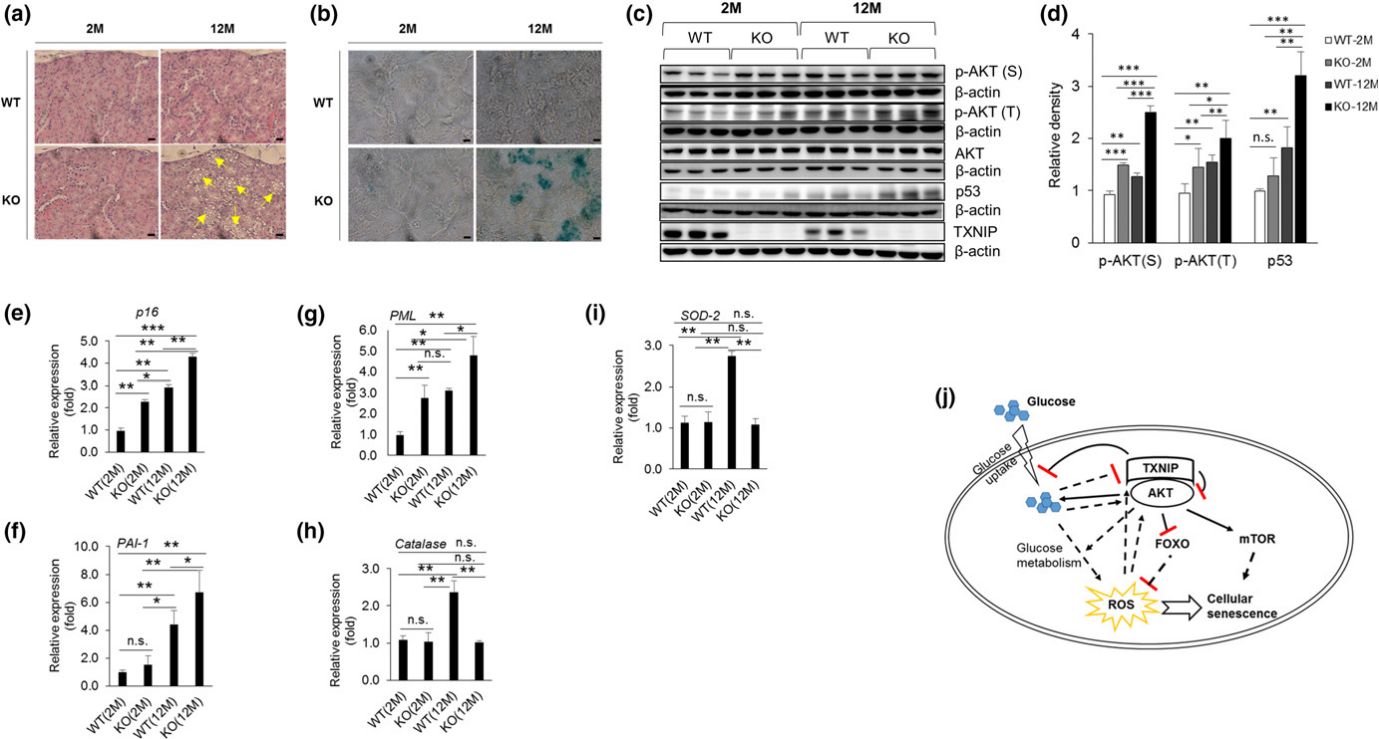
Cellular senescence of KO kidney cells. (a) Representative images of hematoxylin and eosin (H&E) staining of WT and KO kidneys at 2‐month‐old and 12‐month‐old age. Magnification: 400x, scale bar denoted for 20 µm. Yellow arrows indicate degenerated tubular vacuolation. Repeated three times. (b) SA‐β‐gal staining of 12‐month‐old mice kidney samples. Repeated three times. (c, d) Western blotting and statistical analysis of kidney lysates at 12‐month‐old mice. Repeated three times. (e–g) mRNA expression of senescence‐associated genes in WT and KO kidneys (*n* = 4). Repeated two times. Statistical significance was determined using ANOVA. *p < 0.05, **p < 0.01, ***p < 0.001. (h, i) The expression of FOXO target genes. Catalase (h) and SOD2 (i) (*n* = 4). Repeated two times. Statistical significance was determined using ANOVA. *p < 0.05, **p < 0.01, ***p < 0.001. (j) Overview of the regulation of AKT‐mediated cellular senescence by TXNIP. AKT activation induces glucose uptake, which in turn induces cellular senescence by increasing ROS levels via inhibition of the transcriptional activity of the FOXO family and by activating mTOR pathways. On the other hand, TXNIP inhibits AKT activation by direct interaction with AKT or by inhibiting glucose uptake. Consequently, TXNIP regulates AKT‐mediated senescence of cells in vitro and in vivo

One of the organs sensitive to oxidative stress, which increases susceptibility to apoptosis and is associated with delayed repair and regeneration, is the kidney (Yang & Fogo, 2010). Kidney damage is associated with many health problems in the elderly, including renal cancer and kidney failure (Melk et al., 2003). To identify the function of TXNIP in kidney degeneration, we analyzed kidney samples from WT and KO mice at 2, 12, and 20 months of age using hematoxylin and eosin (H&E) staining, SA‐β‐gal staining, or western blotting. Here, 12‐month‐old KO mice showed marked kidney tubular vacuolation in H&E staining (Figure 6a and Supporting Information Figure S4A) and marked SA‐β‐gal staining (Figure 6b and Supporting Information Figure S4B). Furthermore, the 12‐month‐old KO mice exhibited more AKT activation, p53 expression (Figure 6c,d and Supporting Information Figure S5), and increased expression of senescence‐associated genes including p16, PAI‐1, and PML (Figure 6e–g), and showed decreased expression of antioxidant genes, catalase and SOD2 which are upregulated by FOXO transcription factor (Figure 6h,i), compared to the 12‐month‐old WT mice. In addition, to understand the function of TXNIP under normal aging condition, we examined 2‐month‐old (2 M) and 20‐month‐old (20 M) WT and KO mice. Twenty‐month‐old WT mice showed hyperactivation of AKT and induction of TXNIP levels than 2‐month‐old WT mice (Supporting Information Figure S4C). Twenty‐month‐old WT mice showed more glucose tolerance than 2‐month‐old WT mice, and interestingly, 20‐month‐old KO mice showed dramatically decreased blood glucose levels under normal and fasting condition than 2‐month‐old KO mice or other groups (Supporting Information Figure S4D). Next, to investigate the activation of AKT or expression of TXNIP by glucose injection in WT and KO mice, we obtained kidney samples from GTT‐tested mice in Supporting Information Figure S4C. We determined AKT activation and TXNIP expression in WT and KO kidney cells using western blotting after 2 hr of glucose injection. AKT was activated, but the level of TXNIP was decreased by glucose injection (Supporting Information Figure S4E). Taken together, these results suggest that TXNIP regulates cellular senescence by inhibiting the expression of senescence‐associated genes in mice kidney cells.

## 3 DISCUSSION

In this study, we elucidated that TXNIP deficiency accelerated the senescence of MEF cells at late passages in a manner dependent on ROS levels and AKT activity (Figures 1 and 2). TXNIP is a well‐known tumor suppressor and pro‐oxidant protein that inhibits TRX activity via direct interaction with the catalytic active center of TRX (Jung & Choi, 2014). Apart from the tumor‐suppressing function of TXNIP in cancer cells, many reports have suggested that TXNIP regulates glucose homeostasis by inhibiting glucose uptake and glucose metabolism (Chutkow et al., 2008; Parikh et al., 2007; Wu et al., 2013). Here, we examined the induction of glucose uptake in KO MEF cells (Figure 1i) and KO mice (Figure 4a,b, 5a, and Supporting Information Figure S4D). Although previous reports have revealed the role of TXNIP in the regulatory mechanisms of glucose uptake and glucose metabolism in cancer and primary cells, the function of TXNIP in cellular senescence under glucose stress has not been reported (DeBalsi et al., 2014; Hui et al., 2008; Wu et al., 2013). High glucose is a well‐known inducer of cellular ROS and accelerates the senescence of cells (Mortuza et al., 2013). From these reports, we can suggest that excessive or uncontrolled glucose uptake caused by TXNIP deficiency serves as a high energy source in cells, which may consequently induce cellular senescence by ROS production in KO MEF cells.

Previous reports have provided partial support for the activation of AKT in KO MEF cells (Figure 2). AKT plays an essential role in the regulation of glucose uptake and metabolism. AKT regulates the expression and translocation of GLUT4 to induce glucose uptake and is correlated with the regulation of glucose metabolism, and the PI3K/AKT pathway promotes cellular glucose uptake by downregulating the expression of TXNIP (Hajduch et al., 2001; Hong et al., 2016; Tan et al., 2010). KO mice exhibited greater AKT signaling, insulin sensitivity, and glycolysis in oxidative tissues than WT mice, which was associated with impaired mitochondrial fuel oxidation and the accumulation of oxidized PTEN (Hui et al., 2008). Intracellular ROS induce AKT activation, which in turn induces premature senescence by increasing ROS levels via inhibition of the transcriptional activity of the FOXO family and by activating mTOR pathways (Dolado & Nebreda, 2008; Johnson et al., 2013; Sheu et al., 2004; Zhao et al., 2017). Other groups also reported the function of AKT in aging. AKT2 ablation prolonged lifespan and improved cardiac aging through restored FOXO 1‐related autophagy and mitochondrial integrity (Ren et al., 2017), and chronic AKT activation accentuated aging‐induced cardiac hypertrophy and myocardial contractile dysfunction through a loss of autophagic regulation (Hua et al., 2011).

In our previous reports, we found that TXNIP interacts with p53, p38 MAPK, and macrophage migration inhibitory factor (MIF) and regulates their functional activity (Jung et al., 2016, 2013; Kim et al., 2017). TXNIP stabilized p53 protein via direct interaction, and TXNIP acted as a functional switch of p53 to regulate ROS level in hematopoietic cells. TXNIP‐deficient hematopoietic cells were hypersensitive to oxidative stress and showed lower p53 level in young mice; however, p53 level was higher in old mice. The function of p53 was determined by ROS level whether p53 acts as an antioxidant or a pro‐oxidant protein in hematopoietic cells. In this study, p53 level was lower in KO MEF cells at early passage (P1); however, p53 level was higher in late passage (P5) (Figure 1f,g), and p53 was critical for the cellular senescence of KO MEF cells (Supporting Information Figure S1C,D). Overall, we might suggest that p53 was activated by the elevated ROS levels and acted as a pro‐oxidant protein in KO MEF cells at P5.

AKT consists of an N‐terminal PH domain, a KD, and a C‐terminal regulatory region (HM) (C. Parikh et al., 2012). The PH domain is a structurally related regulatory module that is present in a variety of proteins involved in signal transduction (Rameh et al., 1997). PI3K phosphorylates membrane phosphatidylinositol–4,5‐bisphosphate (PtdIns (4,5) P2) to form trisphosphate PtdIns (3,4,5) P3, which binds to the PH domain of AKT, thereby opening the structure of AKT to allow for its activation by its modulators (Mahadevan et al., 2008). The intramolecular PH domain–KD interactions are important in maintaining AKT in an inactive state. AKT activation occurs after a conformational change that dislodges the PH domain from the KD (Parikh et al., 2012). Some previous reports have suggested that the PH domain may interact with other proteins. Protein kinase C interacts with the PH domain of Bruton tyrosine kinase (BTK) and inhibits BTK activity (Yao, Kawakami, & Kawakami, 1994). One group has reported that N‐ribosyldihydronicotinamide:quinone reductase 2 (NQO2), an oxidoreductive enzyme, interacts with AKT via the PH domain of AKT to inhibit AKT activity (Hsieh et al., 2014). Here, we showed that TXNIP interacts with AKT via the PH domain of AKT and inhibits the kinase activity of AKT (Figure 3).

As mentioned above, AKT is an important regulator of glucose uptake and glucose metabolism, and high glucose‐induced AKT activation induces premature senescence by increasing ROS levels. In addition, TXNIP regulates glucose uptake by regulating the glucose transporter. Therefore, we hypothesized that TXNIP and AKT interacted to modulate glucose metabolism or glucose uptake‐driven metabolic stress. In this study, we showed opposite effects on the expression of TXNIP and the activity of AKT under glucose stress (Figure 2). However, the interaction of TXNIP and AKT was upregulated by glucose and H_2_O_2_ treatment (Figure 3), implying that TXNIP may negatively regulate AKT activity under glucose stress and oxidative stress. We previously reported that TXNIP deficiency induced premature aging phenotypes of HSCs in aged mice by increasing ROS levels in vitro and in vivo (Jung et al., 2016, 2013 ). Next, we analyzed the metabolic characterization of WT and KO mice to determine the effect of TXNIP on the aging of mice. TXNIP‐deficient mice exhibited the reduction in energy expenditure in metabolic analysis (Figure 5), as well as the induction of senescence‐associated gene expression and SA‐β‐gal staining in kidney at 12 months of age (Figure 6).

In conclusion, we demonstrated that TXNIP may regulate cellular senescence by inhibiting AKT activity via a direct interaction under glucose stress (Figure 6j). Altogether, our results suggest that the TXNIP‐AKT axis may be a possible therapeutic target for the regulation of cellular senescence.

## 4 EXPERIMENTAL PROCEDURES

### 4.1 Animals

TXNIP^‐/‐^ mice (C57BL/6) were generated and were genotyped as previously reported (Jung et al., 2016). Akita mice were obtained from Dr. Chul‐Ho Lee (KRIBB, Korea). Wild‐type C57BL/6 mice were purchased from Koatech (Pyeongtaek, Korea). To make littermate control, we crossed TXNIP^‐/‐^ mice with WT mice then used for the mice experiments. To determine the genotypes of every mouse in the litter, the primer sequences were used as follows: TXNIP forward 5′‐ ATTCCCCTTCCAGGTGGA‐3′, reverse 5′‐ TTGAAATTGGCTCTGT‐3′ and LacZ forward 5′‐ GAAGCCAATATTGAAACCCA‐3′, reverse 5′‐ GCAAAGACCAGACCGTTCAT‐3′. In brief, we followed these PCR protocols: for TXNIP, 94°C‐5 min/1 cycle, 94°C ‐1 min, 55°C‐1 min, 72°C‐1 min/35 cycles, 72°C‐7 min/1 cycle, 4°C; and for LacZ, 94°C‐5 min/1 cycle, 94°C‐1 min, 55°C‐1 min, 72°C‐1 min/30 cycles, 72°C‐7 min/1 cycle, 4°C (Lee et al., 2005). We crossed TXNIP^‐/‐^ mice with Akita^±and^ TXNIP/Akita^±mice^ are used in Figure 4. Male mice were used for overall study, and all mice were housed in a pathogen‐free animal facility under a 12‐hr light–dark cycle. All animal experiments were approved by the Institutional Animal Use and Care Committee of the Korea Research Institute of Bioscience and Biotechnology (KRIBB) and were performed in accordance with the Guide for the Care and Use of Laboratory Animals published by the US National Institutes of Health.

### 4.2 SA‐β‐gal and hematoxylin and eosin staining

For SA‐β‐gal staining, freshly isolated kidneys were immediately embedded in frozen section compound FSC22 (Leica Biosystems) and kept at −80°C. The samples were subjected for cryosection within 24 hr, and 5‐μm‐cryosectioned tissues were directly stained with SA‐β‐gal staining kit (9,860, Cell Signaling Technology) according to the manufacturer's instruction. Plus, 10X staining solution was diluted 1:10 with sterile distilled water (pH 6). Blue color cells were detected under a bright field microscope (Olympus IX53; Olympus) at 400X total magnification. For H&E staining, kidney samples were fixed in 4% formaldehyde and embedded in paraffin wax. Sectioned tissues were countered staining with Mayer's Hematoxylin (Dako, Carpinteria, CA) and ClearView Eosin (BBC Biochemical, Mt. Vernon, WA) and detected as described above.

### 4.3 Histopathologic evaluation of tubular vacuolation

H&E‐stained kidney samples were taken a picture at magnification 400X. In this study, scoring of renal tubular vacuolation was based on the following criteria: 0%—normal (0), <10%—minimal (1), 10%–30%—mild (2), 31%–50%—moderate (3), and >51%—severe (4). We considered the presence of clear vacuoles in renal tubule, and the distribution of tubular vacuolation was graded (Bendele, Seely, Richey, Sennello, & Shopp, 1998).

### 4.4 Statistical analysis

Prism 5.00 software (GraphPad Software, Inc.) was used for data analysis. To quantify the expression of protein, we used CS Analyzer 4 software (Atto, Japan). Data are expressed as the mean ± *SD* of *n* determinations and statistical significance was determined using Student's *t* tests, unless mentioned differently. *p < 0.05, **p < 0.01, ***p < 0.001. *P* value <0.05 was considered to represent a significant difference. For animal studies in Figure 6, statistical significance was determined using ANOVA. *p < 0.05, **p < 0.01, ***p < 0.001.

## CONFLICT OF INTEREST

The authors declare no competing financial interests.

## AUTHOR CONTRIBUTIONS

H.H. and H.Y.S. designed and performed experiments and analyzed the data. M.J.K. performed GST pull‐down assay and a proximity ligation assay. W.S.K. performed kinase assay. D.O.K. and J.‐E.B. performed mice experiments. J.L. and E.‐J.C. performed flow cytometry analysis and plasmid construction. Y.‐J.P., T.‐D.K., S.R.Y., and J.‐Y.N. provided helpful discussions and crucial analysis of data. C.‐H.L. performed mice management. I.C. and H.J. supervised the overall project, analyzed the data, and wrote the manuscript.
