# Supplementary figures and images for: TXNIP regulates AKT‐mediated cellular senescence by direct interaction under glucose‐mediated metabolic stress
Source: Aging Cell. 2018 Aug 31;17(6):e12836. doi: 10.1111/acel.12836 (PMC6260918; doi:10.1111/acel.12836)

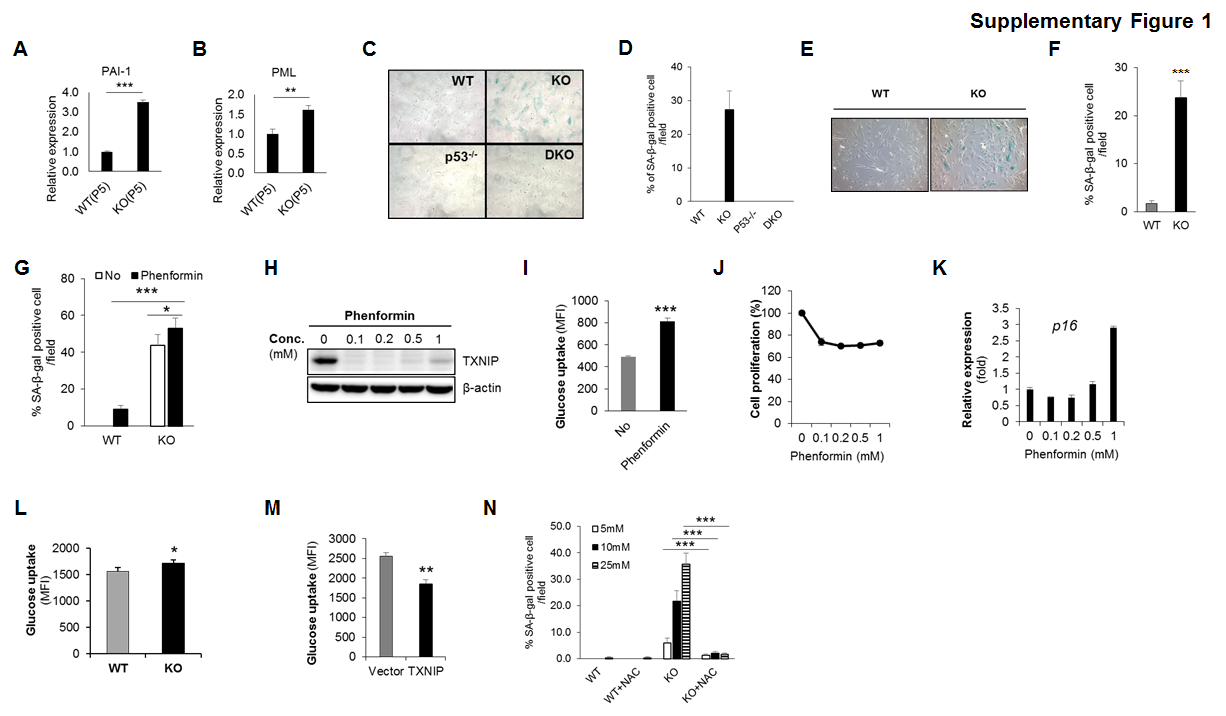

Supplement: Supplementary file 1 [file ACEL-17-e12836-s001.tif]

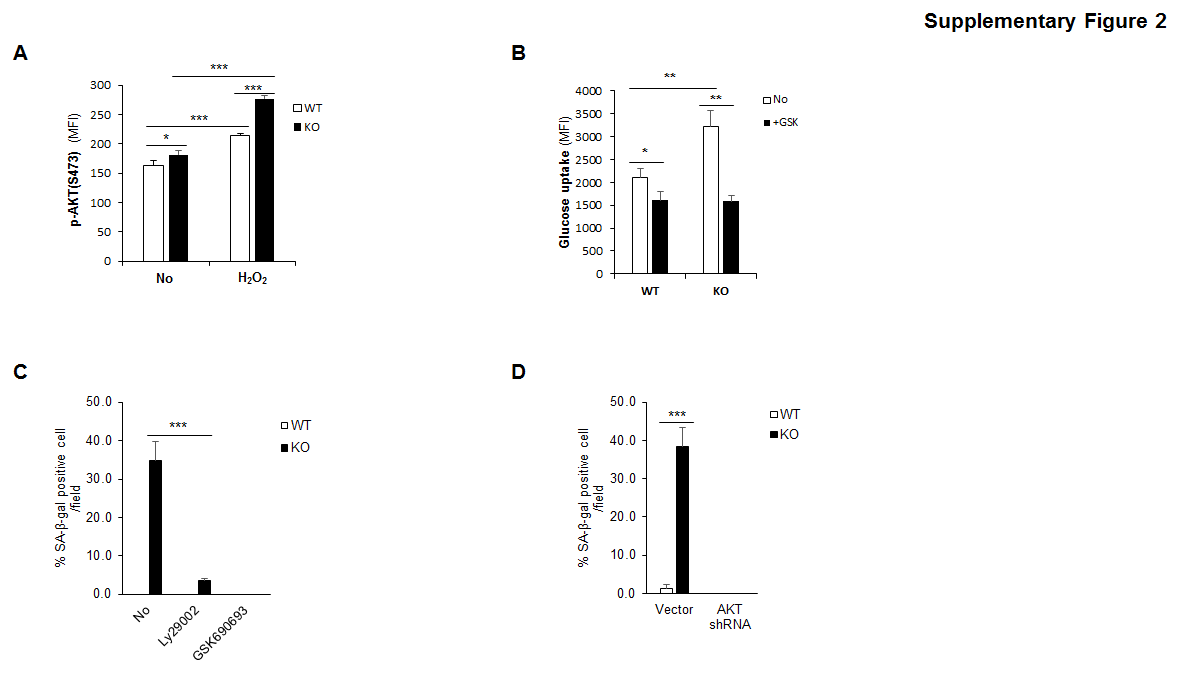

Supplement: Supplementary file 2 [file ACEL-17-e12836-s002.tif]

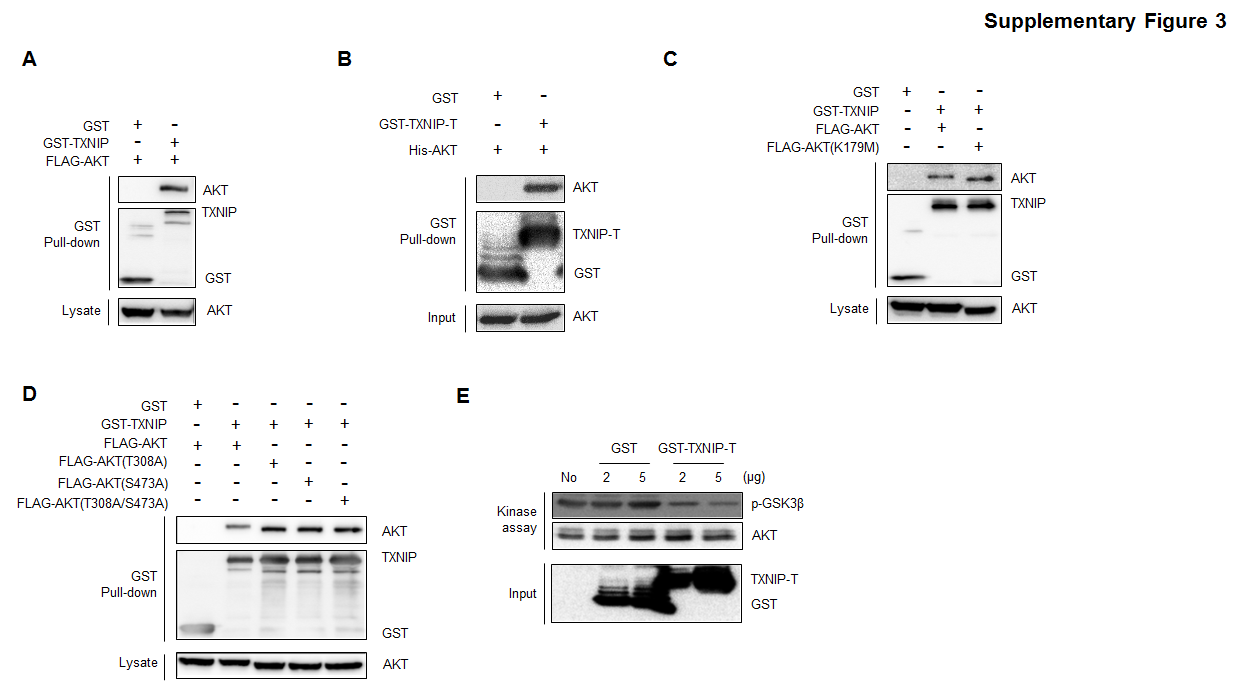

Supplement: Supplementary file 3 [file ACEL-17-e12836-s003.tif]

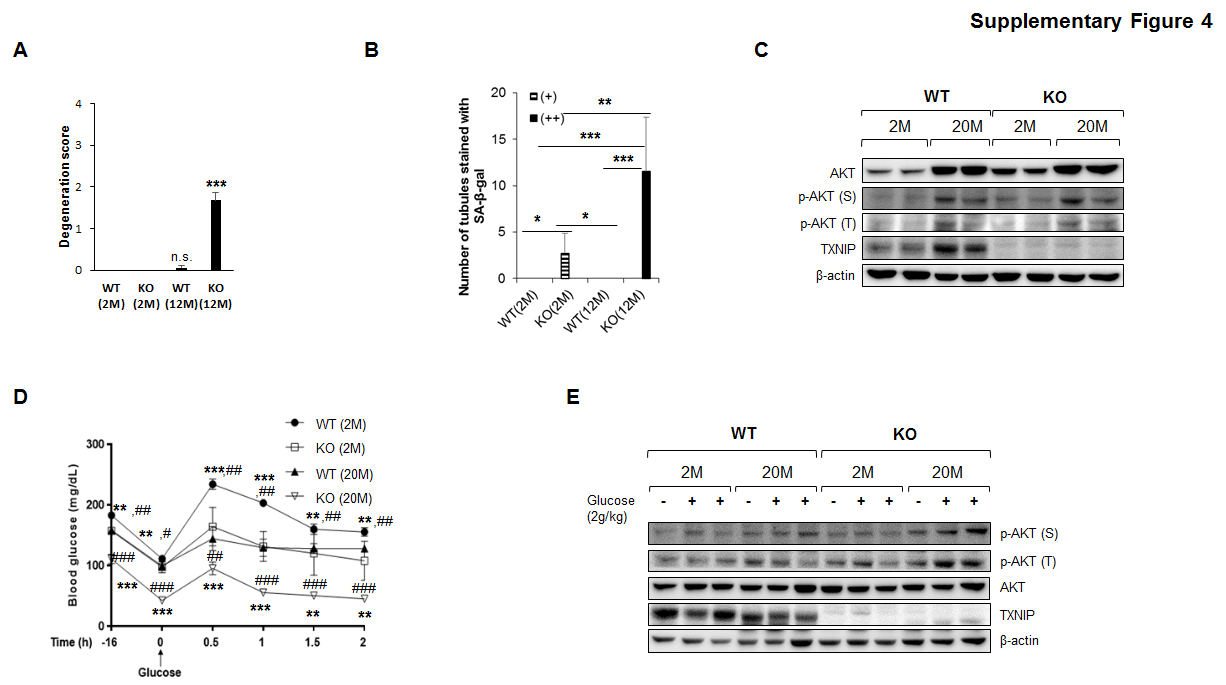

Supplement: Supplementary file 4 [file ACEL-17-e12836-s004.tif]

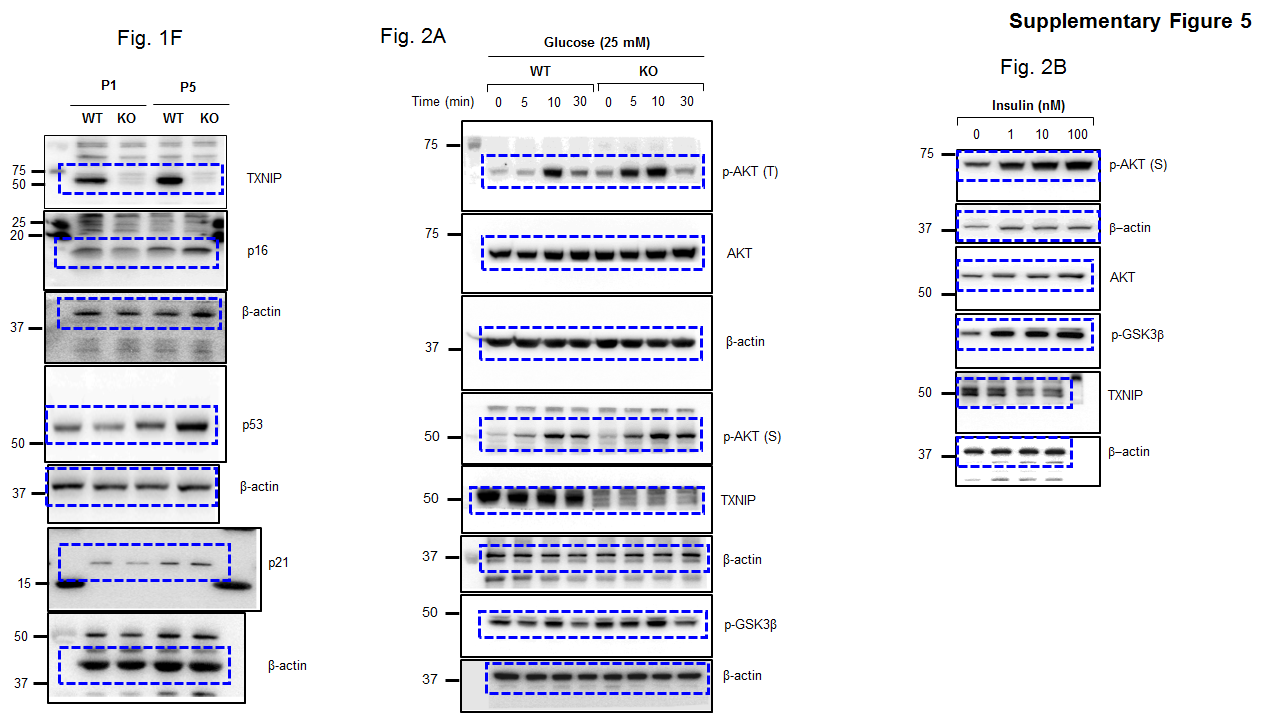

Supplement: Supplementary file 5 [file ACEL-17-e12836-s005.tif]

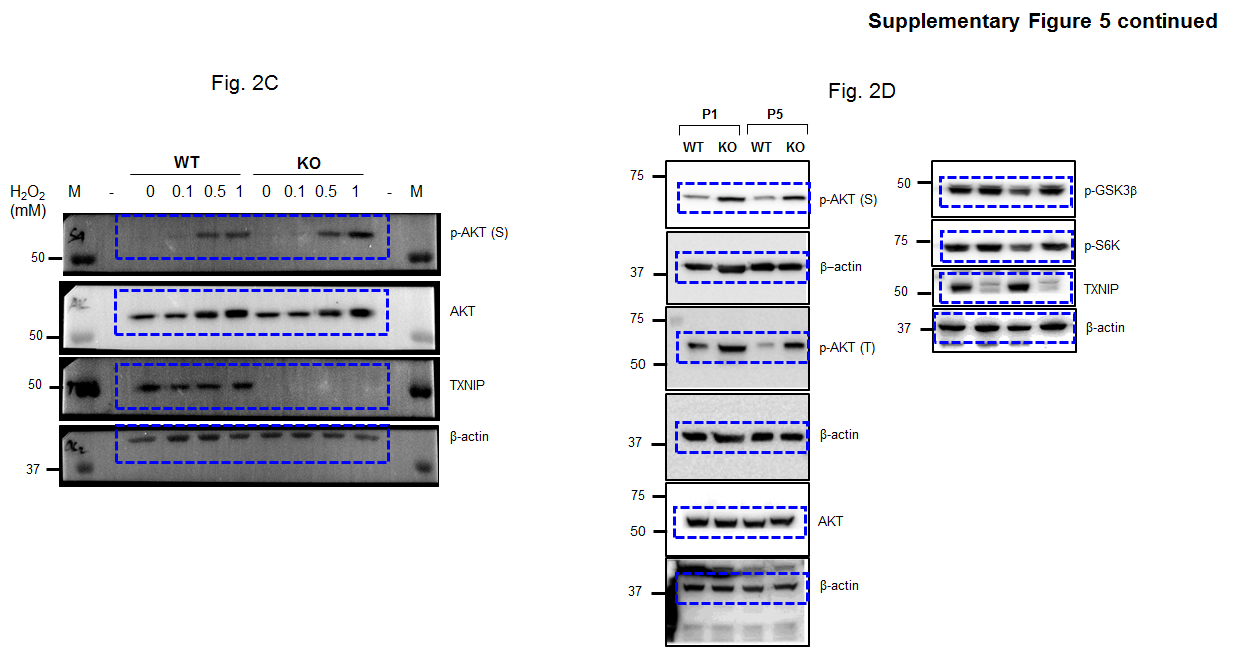

Supplement: Supplementary file 6 [file ACEL-17-e12836-s006.tif]

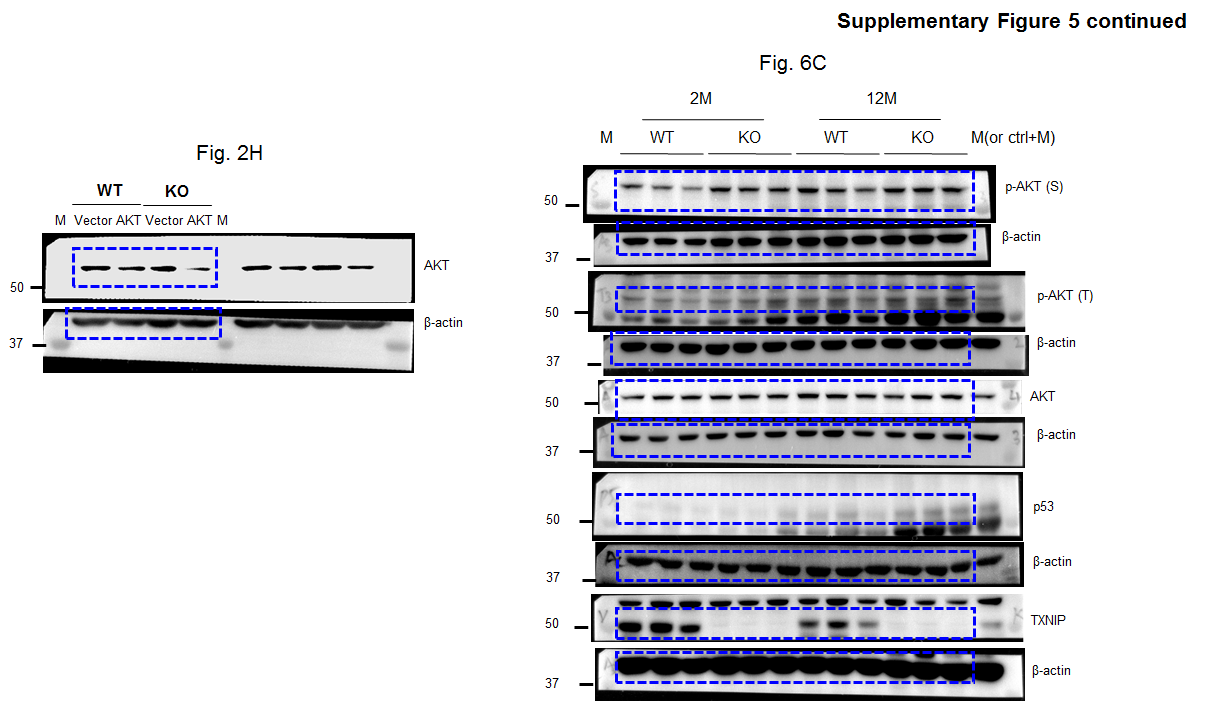

Supplement: Supplementary file 7 [file ACEL-17-e12836-s007.tif]
