# Supplementary material for: TXNIP regulates AKT‐mediated cellular senescence by direct interaction under glucose‐mediated metabolic stress
Source: Aging Cell. 2018 Aug 31;17(6):e12836. doi: 10.1111/acel.12836 (PMC6260918; doi:10.1111/acel.12836)
Supplement: Supplementary file 8 [file ACEL-17-e12836-s008.docx]

**Supplemental Experimental Procedures**

**Metabolic analysis**

For metabolic analysis, oxygen consumption (VO2), carbon dioxide production (VCO2), energy expenditure (EE), and physical activity were measured using the OxyletPro Physiocage System (Panlab/Harvard apparatus, Cornella, Spain) and OxyletPro METABOLISM (V3.0.00, Panlab). The respiratory exchange ratio (RER) was calculated as VCO_2_/VO_2_. Mice were placed to the metabolic chambers for 2 days then used second day results for metabolic analysis.

**Blood glucose detection and mice survival**

Blood glucose levels of wide type and TXNIP knockout mice were detected in the morning using Accu-Check Active Strip and Accu-Check Active blood glucose meter (Roche, Mannheim, Germany). For mice survival results, Akita and KO/Akita mice were monitored daily and updated every week.

**Cell culture**

Mouse embryonic fibroblast (MEF) cells were isolated from pregnant mice at 13.5 days of gestation and mouse primary lung fibroblast cells isolated from 8-12-week-old male C57BL/6 mice. 293T cells were obtained from the American Type Culture Collect (Manassas, VA). All cells were maintained in DMEM (WELGENE Inc., Gyeongsan, Korea) supplemented with 10% fetal bovine serum (Gibco) and antibiotic-antimycotic (Gibco) or gentamycin (Gibco) at 37ºC and 5% CO_2_.

**Reagents**

Ly294002 (S1105) and GSK690693 (S1113) were acquired from Selleckchem. D-(+)-Glucose (G6152), Hydrogen peroxide (323381), *N*-Acetyl-L-cysteine (A7250) and Phenformin HCL (P7045) were purchased from Sigma-Aldrich.

**Quantitative Real-Time PCR**

The total RNA of cells or tissues were isolated using TRIzol (Qiagen). 1 μg of each sample were subjected for the reverse transcriptase PCR by First-Strand cDNA synthesis kit (FSK-101, Toyobo). cDNAs were diluted 1:10 and submitted to quantitative PCR using the SYBR Premix ExTaq (Takara Bio) and Applied Biosystems ViiA 7 Real-Time PCR system (Thermo Fisher Scientific). The primer sequences were as follows: mouse 18S RNA forward 5’-GCAATTATTCCCGAACG-3’ and reverse 5’-GGCCTCACTAAACCATCCAA-3’, mouse p16 forward 5’-CGAACTCGAGGAGAGCCATC-3’ and reverse 5’-TACGTGAACGTTGCCCATCA-3’, mouse catalase forward 5’-AAGATTGCCTTCTCCGGGTG-3’ and reverse 5’-TGTGGAGAATCGAACGGCAA-3’, mouse SOD-2 forward 5’-AGGAGAAGTACCACGAGGCT-3’ and reverse 5’-GCAGGCAGCAATCTGTAAGC-3’, mouse foxo3a forward 5’-GAGCTGGAGCTCGAACCTT-3’ and reverse 5’-CTTCATCGTCGTCCTCCTCG-3’, mouse PML forward 5’-AACAGAGGAGCGAGTTGTGG-3’ and reverse 5’-CCAGCTGGCTAATTTTCTGGG-3’, and mouse PAI-1 forward 5’-CCGATGGGCTCGAGTATGAC-3’ and revers 5’-TTCTCAAAGGGTGCAGCGAT-3’

**Intracellular staining and confocal imaging**

For phospho-AKT analysis using flow cytometry, we treated WT and KO MEF cells with H_2_O_2_ (1 mM) for 1 hour. Trysinized MEF cells were permeabilized with the Cytofix/Perm Solution (BD Biosciences). For phopho-AKT staining, the cells were blocked with PBS containing 5% BSA for 30 mins at RT and then incubated with primary antibodies for 1 h in PBS containing 5% BSA, washed 3 times with PBS, and then incubated with anti-phospho-AKT (560404, BD Biosciences) or anti-mouse IgG1 (553443, BD Biosciences) at 4℃ for 30 min in PBS containing 2% FBS. Stained cells were analyzed using FACSCanto II (BD Biosciences). For confocal imaging, WT and KO MEF cells were seeded on coverslips in the cell culture plates. After 1 day, cells were washed twice with cold PBS then immediately fixed. After fixation, cells were permeabilized with 0.2% Triton X-100 for 20 min and blocked with PBS containing 5% BSA for 30 min at RT. Cells were stained with phospho-H2AX (Ser 139) antibody (2577, Cell Signaling) and secondary Alexa Fluor 488 antibody (Life technology) for 1 hour at RT and then were mounted with DAPI containing mounting reagent (Molecular Probes). The images were captured using a LSM800 confocal microscope (Carl Zeiss).

**Western blotting and Immunoprecipitation**

The proteins were extracted from cells using buffer containing 0.1% NP40, 0.3 mM NaCl, 20 mM HEPES (pH 7.2), 0.1 M KCl, 10 mM EDTA, 1 mM Na3VO4, 10 mM NaF, 25 mM sodium β-glycerophosphate, and 2 mM sodium pyrophosphate. Inhibitors for protease and phosphatase (Calbiochem, San Diego, CA) were also added to prevent the enzymatic reaction (M. J. Kim et al., 2017; W. S. Kim et al., 2017). All lysates were quantified and loaded the same amount of protein on the 8-12% SDS-PAGE gel before transferring to the PVDF membrane (Millipore, Bedford, MA). The primary antibodies acquired from Cell Signaling Technology were AKT, p-AKT (S473), p-AKT (T308), p-GSK-3β (S9), p-S6K (S235/236), TXNIP, Foxo3a, and p-Foxo3a; and from Santa Cruz Biotechnology, p16, p21, SOD-2, Catalase, and β-actin. After incubation with peroxidase-conjugated anti-mouse or anti-rabbit IgG (Jackson ImmunoResearch), proteins were detected using EzWestLumiOne (ATTO, Tokyo) or SuperSignal West Pico Chemilumicesent (Thermo Scientific) substrate and WSE-6200 LuminoGraph II Imaging System (ATTO, Tokyo). For exogenous immunoprecipitation assay, the lysate proteins of co-transfected cells were incubated with Glutathione Resin (L00206, GeneScript) for 3 hours rocking at 4ºC. Beads were then washed five times with lysis buffer and loaded on the SDS-PAGE gel. Western blot was performed as described above with the primary and anti-FLAG (Sigma aldrich) or anti-GST antibodies (Santa Cruz Biotechnology). For endogenous binding of TXNIP and AKT, the lysate proteins were pre-cleared with Protein A/G PLUS-Agarose (Santa Cruz Biotechnology) for 30 minutes rocking at 4ºC. Then lysates were incubated for 1 hour at 4ºC with 5 μg of anti-TXNIP antibody or IgG control antibody (Santa Cruz) before another 2 hours incubation with Protein A/G PLUS-Agarose. The precipitations were washed five times with lysis buffer. The bound proteins were visualized by Western blot with anti-AKT antibody.

**shRNA transduction**

Retroviral pRS vector or pRS AKT shRNA (Origene) was generated with the Platinum-E retroviral packaging cell line (CellBiolabs). P1 MEF cells were infected by each retrovirus in DMEM (WELGENE Inc., Gyeongsan, Korea) supplemented with 10% fetal bovine serum (Gibco) two times for 2 days incubation. After 48 h incubation, transduced cells were harvested and submitted to Western blot analysis to confirm knockdown of AKT in MEF cells or were subcultured to P5.

**Reactive oxygen species (ROS) analysis**

Cellular ROS contents were measured by incubating cells with 5 μM of CM-H_2_DCFDA (Thermo Fisher Scientific) for 20 minutes and analyzed within 1 hours after incubation by flow cytometry (BD FACS Canto II). Data were expressed as the value of the mean of fluorescence intensity (MFI) (Jung et al., 2016; Jung et al., 2013).

***In vitro* binding and kinase assay**

Full-length TXNIP recombinant was insoluble. Therefore, we prepared GST and GST-fused truncation mutant for TXNIP (150a.a-317a.a) (GST-TXNIP-T) and were purified as described in our previous report (Jung *et al.* 2016). His-AKT1 was acquired from Thermo Fisher scientific (P2999). To perform GST pull-down assay, we mixed GST (1-2 µg) with His-AKT (1-2 µg) or GST-TXNIP-T (1-2 µg) with His-AKT (1-2 µg) in lysis buffer. Protein mixtures were incubated with Glutathione Resin (L00206, GeneScript) for 3 hours rocking at 4ºC. Beads were then washed five times with lysis buffer and loaded on the SDS-PAGE gel. Western blot was performed with the primary and anti-AKT or anti-GST antibodies (Santa Cruz Biotechnology). To examine the kinase activity of AKT *in vitro*, we used AKT kinase assay kit (9840, Cell Signaling). To prepare active AKT, we immunoprecipitated AKT from 293T cell lysate (500 ug/each) using anti-AKT antibody (9272, Cell Signaling). The lysate proteins were pre-cleared with Protein A/G PLUS-Agarose (Santa Cruz Biotechnology) for 30 minutes rocking at 4ºC. Then lysates were incubated for 1 hour at 4ºC with 5 μg of anti-AKT antibody before another 2 hours incubation with Protein A/G PLUS-Agarose. The precipitations were washed two times with lysis buffer. We added 2, and 5 µg of GST or GST-TXNIP-T (150-317) into AKT precipitations with GSK3β substrate. We followed manufacturer’s instruction and AKT kinase activity was determined by phospho- GSK3β.

**Confocal imaging and Proximity ligation assay (PLA)**

MEF cells were fixed and permeabilized with the Cytofix/Perm Solution (BD Biosciences). The cells were incubated with primary antibodies for 1 h in PBS containing 2% FBS, washed 3 times with PBS, and then incubated with a secondary Alexa Fluor 488 or 546 (Invitrogen). The images were captured using a LSM510 confocal microscope (Carl Zeiss). For in situ Proximity ligation assay (PLA)**,** MEF cells were plated on round glass cover slips in 6-well plates 24h, and then the cells were stimulated with glucose (25 mM) for 1h. Cells were washed twice with cold PBS and fixed for 20 min at room temperature with 4% paraformaldehyde in PBS, followed by permeabilization with 0.2% Triton X-100. Proximity ligation assay performed according to instructions supplied with Duolink In situ kit (Olink Bioscience, Uppsala, Sweden) as described previous report (Jung et al., 2016; M. J. Kim et al., 2017).

**Supplemental Figure Legends**

**Fig. S1. Cellular senescence and glucose uptake regulation** (A,B) Expression of senescence-associated genes in P5 of WT and KO MEF cells (n=4). (C,D) p53 deficiency in KO MEF cells completely blocks SA-β-gal staining at P5. Repeated 2 times (n=4). (E,F) SA-β-gal staining of KO lung fibroblast cells at P5. Mouse primary lung fibroblast cells were subcultured from P1 to P5 and stained with SA-β-gal at P5. (G) SA-β-gal staining of MEF cells. Phenformin induces SA-β-gal staining of WT MEF cells. Related to Fig. 1G. (H) TXNIP is dramatically decreased by Phenformin treatment in P1 MEF cells. P1 MEF cells were treated with various doses of Phenformin. (I-K) Phenformin induces glucose uptake (n=4) (I), decreases the cell proliferation (n=3) (J), and induces p16 (n=3) (K). (L) Glucose uptake in P1 MEF cells (n=4). (M) Regulation of glucose uptake by TXNIP overexpression in MEF cells (n=4). WT MEF cells were transfected with FLAG vector or FLAG-TXNIP. After 48 hours, glucose uptake was measured by flow cytometry. (N) SA-β-gal staining of MEF cells. Related to Fig. 1J. Data are mean ± s.d. Statistical significance was determined using Student’s *t*-tests. **P*< 0.05, ***P*< 0.01, ****P*<0.001.

**Fig. S2. Regulation of cellular senescence.** (A) Differential activation of AKT in KO MEF cells by H_2_O_2_ treatment. MEF cells were treated with H2O2 (1 mM) for 30 minutes. Related to Fig. 2C (n=3). Repeated 3 times. (B) Regulation of glucose uptake by AKT inhibition. MEF cells were treated with GSK690693 (5 µM), an AKT inhibitor for 40 minutes. (C) % SA-β-gal staining of MEF cells. Related to Fig. 2F. (D) % SA-β-gal staining of MEF cells. Related to Fig. 2G. Data are mean ± s.d. Statistical significance was determined using Student’s *t*-tests. **P*< 0.05, ***P*< 0.01, ****P*<0.001.

**Fig. S3. TXNIP interacts with AKT and regulates kinase activity.** (A) GST pull-down assay between TXNIP and AKT in 293T cells. (B) Recombinant binding assay. To perform GST pull-down assay, we mixed GST (1-2 µg) with His-AKT (1-2 µg) or GST-TXNIP-T (1-2 µg) with His-AKT (1-2 µg) in lysis buffer. Repeated 2 times. (C) GST pull-down assay between TXNIP and kinase dead mutants of AKT. (D) TXNIP interacts with AKT mutants with active phosphorylation sites (T308A, S473A or T308A/S473A). (E) In vitro kinase assay. We immunoprecipitated AKT from 293T cell lysate (500 µg/each) using anti-AKT antibody then added recombinant GST or GST-TXNIP-T fusion protein ( 2 and 5 µg ) as indicated. Repeated 2 times.

**Fig. S4. Glucose uptake induction and AKT activation in aged mice.** (A) Distribution of renal tubular vacuolation was graded. (n=7-9) Repeated 3 times. To score the tubular vacuolation, we graded cytoplasmic vacuolation as % area portion in 400X magnification picture. Statistical significance was determined using ANOVA. ****P*<0.001. (B) SA-β-gal staining of kidney cells. Related to Fig. 6B (n=7-9). Statistical significance was determined using ANOVA. **P*< 0.05, ***P*< 0.01, ****P*<0.001. Repeated 3 times. (C) Regulation of TXNIP and AKT activation with age. Repeated 3 times. (D) GTT test (n=5-7). Repeated 2 times. **P* compared to same genotype mice and different age and ^#^*P* compared to different genotype and same age. * and ^#^ indicate significant difference between indicated two groups. **P*< 0.05, ***P*< 0.01, ****P*<0.001. Statistical significance was determined using Student’s *t*-tests. (E) Western blotting image for GTT tested kidney samples. Glucose no treated (-) samples are 18 hours fasted samples and glucose injected samples are sacrificed after 2 hours injection. Repeated 2 times.

**Fig. S5. Uncropped, full Western blot images of important results in indicated figures (Fig. 1F, Fig. 2A, 2B, 2C, 2D, 2H and Fig. 6C).**
